# Prevalence and Predictors of Self-Reported Sexual Abuse in Severely Obese Patients in a Population-Based Bariatric Program

**DOI:** 10.1155/2013/374050

**Published:** 2013-06-23

**Authors:** Danielle L. Gabert, Sumit R. Majumdar, Arya M. Sharma, Christian F. Rueda-Clausen, Scott W. Klarenbach, Daniel W. Birch, Shahzeer Karmali, Linda McCargar, Konrad Fassbender, Raj S. Padwal

**Affiliations:** ^1^Department of Medicine, University of Alberta, Edmonton, AB, Canada T6G 2B7; ^2^Alberta Diabetes Institute, Edmonton, AB, Canada T6G 2B7; ^3^Department of Surgery and CAMIS (Center for the Advancement of Minimally Invasive Surgery), University of Alberta, Royal Alexandra Hospital, Edmonton, AB, Canada T5H 3V9; ^4^Department of Agricultural, Food and Nutritional Sciences, University of Alberta, Edmonton, AB, Canada T6G 2R3; ^5^Department of Oncology, University of Alberta, Edmonton, AB, Canada T6G 2G2; ^6^Walter C. Mackenzie Health Sciences Centre, 8440-112th Street, Edmonton, AB, Canada T6G 2B7

## Abstract

*Background*. Sexual abuse may be associated with poorer weight loss outcomes following bariatric treatment. Identifying predictors of abuse would enable focused screening and may increase weight management success. *Methods*. We analyzed data from 500 consecutively recruited obese subjects from a population-based, regional bariatric program. The prevalence of self-reported sexual abuse was ascertained using a single interview question. Health status was measured using a visual analogue scale (VAS). Multivariable logistic regression was performed to identify sexual abuse predictors. * Results*. The mean age was 43.7 y (SD 9.6), 441 (88.2%) were females, 458 (91.8%) were white, and the mean body mass index (BMI) was 47.9 kg/m^2^ (SD 8.1). The self-reported prevalence of past abuse was 21.8% (95% CI 18.4–25.4%). Abused subjects had worse health status (VAS score 53.1 (SD 21.2) versus 58.0 (SD 20.1), *P* = 0.03). BMI was not associated with abuse (*P* > 0.5). Age, sex, BMI, and covariate-adjusted independent predictors of abuse included alcohol addiction (adjusted odds ratio 15.8; 95% CI 4.0–62.8), posttraumatic stress disorder (4.9; 2.5–9.5), borderline personality (3.8; 1.0–13.8), depression (2.4; 1.3–4.3), and lower household income (3.4; 1.6–7.0). *Conclusions*. Abuse was common amongst obese patients managed in a population-based bariatric program; alcohol addiction, psychiatric comorbidities, and low-income status were highly associated with sexual abuse.

## 1. Introduction

Moderate-to-severe obesity (body mass index (BMI) of ≥35 kg/m^2^) affects 9% of Canadians and has increased in prevalence by 400% over the last two decades [1]. It shortens life expectancy, substantially reduces quality of life, and is debilitating and costly [2]. Bariatric surgery is the most effective treatment currently available for medically refractory obesity and is indicated in patients with BMI levels of ≥40 kg/m^2^ or BMI levels of 35.0–39.9 kg/m^2^ and an obesity-related comorbidity [2]. Bariatric surgery is associated with reductions in mortality and morbidity as well as increases in health-related quality of life [3].

There is an association between sexual abuse and obesity [4, 5]; those with a history of sexual abuse are at a 40–60% increased risk of having a BMI > 35 kg/m^2^ compared to those without such a history [6]. Furthermore, extremely obese individuals reporting a sexual abuse history are also more likely to report psychosocial issues and psychiatric medication use [7]. As obesity severity increases, the self-reported prevalence of sexual abuse as well as other psychosocial issues rises, although this is not a consistent finding [8–11]. Previous studies examining the prevalence of sexual abuse in severely obese patients seeking bariatric care have reported a 16–32% prevalence of sexual abuse [12, 13], compared to approximately 4–20% in the general population [14–16]. A recent meta-analysis of childhood sexual abuse with 10 million subjects estimates the worldwide prevalence to be 13% [17]; similarly, Canadian studies suggest that self-reported sexual abuse during childhood and adolescence occurred in 4% of males and 13% of females [16].

Sexual abuse is important to identify in all at-risk populations, but particularly so in patients seeking bariatric treatment because it has been (albeit inconsistently) associated with poorer weight loss outcomes [12]. Some have theorized that previously abused subjects may view weight as a protective factor, and therefore either sabotage weight loss efforts or being at risk of increased levels of stress and psychopathology out of fear that latent abuse-related experiences may resurface following successful weight reduction [18, 19]. Identifying patients who have experienced sexual abuse may improve their chances of successful weight management by enabling supportive counseling and other treatments to be administered prior to or in conjunction with medical and/or surgical bariatric treatment [20]. Thus, the objectives of this study were to determine the prevalence of sexual abuse in severely obese patients enrolled in a population-based regional obesity program; characterize abused patients in comparison to those not abused; and identify independent predictors of self-reported sexual abuse to better understand the profiles of these patients.

## 2. Methods

### 2.1. Subjects and Setting

Subjects were recruited from the Edmonton Weight Wise regional obesity program for adults. Edmonton Weight Wise was established in 2005 to deliver integrated, patient-focused, evidence-based care to the Edmonton Zone of Alberta Health Services (AHS). The Edmonton Zone is one of the largest integrated health delivery regions in Canada, serving a catchment population of approximately 1.6 million residents within greater Edmonton. Weight Wise consists of a central, regionwide, single-point-of-access referral system; community education and weight management sessions; and a bariatric specialty clinic. The clinic provides both medical and surgical treatment to practitioner-referred patients of 18 years of age or greater with BMI levels of ≥35 kg/m^2^ who have been unsuccessful with prior attempts at managing chronic obesity. Not all patients seek surgery—some are referred for intensive medical management alone. At the time the study was conducted, wait-times to enter the program were over 2 years; 800 new referrals were seen yearly; and 200 bariatric surgeries were performed annually.

### 2.2. Study Cohort

In this cross-sectional analysis, baseline data from five hundred consecutive, consenting adult (age ≥18 years) subjects recruited from Edmonton Weight Wise and comprising the Alberta Population-based Prospective Evaluation of the Quality of Life and Economic Impact of Bariatric Surgery (APPLES) cohort were examined. Details of the APPLES study, including the design and analytic plan, have been previously published [21]. In brief, APPLES is a 500-patient, population-based, two-year prospective controlled naturalistic study designed to assess the impact of extended wait-times on bariatric care and to examine the clinical, humanistic, and economic consequences of bariatric treatment in the Canadian context. Two hundred medically treated (enrolled at the point of initiation of intensive medical treatment), 150 surgically treated (enrolled after approval and just prior to surgery), and 150 wait-listed (facing wait times of ≥2 years to enter Weight Wise) subjects were enrolled between January 2009 and February 2010. Enrolment in the medical arm was greater as a number of these patients were expected to cross over to surgery within the two-year period. The study was approved by the University of Alberta Research Ethics Board, and informed consent was obtained from all subjects. Of eligible patients contacted, 75% agreed to enter the study.

### 2.3. Data Collection

#### 2.3.1. Sexual Abuse

Self-reported sexual abuse was assessed during a private, in-person interview and as part of baseline data collection by asking subjects, “Do you have a history of sexual abuse, in the past or currently?” Hereafter, we refer to a positive answer to this item as self-reported sexual abuse, and this included any lifetime incident perceived by the patient as sexually abusive including a sexual attack. All patients received both verbal and written explanation that their answers were confidential and would not affect their status in the clinic nor their eligibility for bariatric treatments, including surgery (answers remained part of the research data collection and remained separate from the clinical record). None of the patients included in the study reported current sexual abuse.

### 2.4. Other Data Elements

We collected basic sociodemographic information and clinical data. Subjects were asked to rate their overall state of health from 0 to 100 using a visual analogue scale (VAS) with 100 reflecting the “best imaginable state of health” [21]. Body weight was measured using two validated, calibrated bariatric scales to the nearest 0.1 kilogram, with the subject wearing light indoor clothing with empty pockets, no shoes and an empty bladder. Height was measured to the nearest 0.1 cm using a wall-mounted stadiometer.

Subjects were considered hypertensive if they self-reported hypertension, if they were receiving treatment with antihypertensive medication, or if their screening blood pressure was ≥140/90 mm Hg (or ≥130/80 mm Hg in patients with diabetes). The diagnosis of diabetes, dyslipidemia, depression, and other psychiatric disorders was based on self-report or medication usage. The presence of all other comorbidities, including alcohol abuse and personality disorders, was determined by self-report.

### 2.5. Statistical Analyses

Baseline data from all 500 APPLES subjects were combined for the purposes of this analysis. Descriptive analyses, consisting of means, medians, and proportions, were first conducted and the prevalence of sexual abuse was calculated. Baseline characteristics were compared using *t*-tests for continuous variables or chi-square tests for categorical variables. Multivariable binary logistic regression was then performed to identify independent predictors of sexual abuse. Age, sex, and BMI (per unit increase) were forced into all models a priori. Additional potential covariates (coded in the form that they are presented in Table 1) with *P* values < 0.2 on bivariable analyses were entered into the initial model. Study arm (medical/surgical/wait-listed) was also included as a categorical variable in the initial model. The final model was then created using a stepwise backwards selection method using a Wald chi-square *P* value of 0.05 as the threshold for inclusion. All data were complete except for 20 subjects (4%) with missing annual household income. Because socioeconomic data are typically missing not at random [22], we did not impute these data. Instead, a separate indicator variable in the model was used. Data were complete for all other variables. SAS (Version 9.3, Cary, NC, USA) was used for all analyses.

## 3. Results

### 3.1. Baseline Characteristics and Prevalence of Sexual Abuse

Of the 500 patients enrolled, 459 (91.8%) were white and 441 (88.2%) were females (Table 1). The mean age was 43.7 years (SD 9.6), and the mean BMI was 47.9 kg/m^2^ (SD 8.1). Sexual abuse was reported by 109 (21.8%; 95% CI 18.2–25.4%) patients: 104 women (23.6%, 95% CI 19.6–27.6) and 5 men (8.5%, 95% CI 1.1–15.8).

### 3.2. Characteristics Associated with Sexual Abuse

Abused and nonabused patients had similar age and BMI distributions; however, abuse was more common in females compared to males (23.6% versus 8.5%; *P* = 0.008). In addition, abuse was more common in unmarried versus married patients (25.6% versus 19.0%; *P* = 0.08) and in patients with an annual household income less than $30,000 compared to those with an annual income of $80,000 or higher (41.2% versus 12.3%  *P* < 0.0001) (Table 1). Furthermore, compared to nonabused patients, those who reported sexual abuse were also more likely (*P* < 0.0001) to report depression (83.5% versus 58.3%), bipolar and psychotic illness (14.7% versus 3.3%), posttraumatic stress disorder (32.1% versus 5.6%), binge eating disorder (40.4% versus 26.6%), attention deficit disorder (23.9% versus 8.7%), addiction to alcohol (11.9% versus 0.8%) or drugs (9.2% versus 2.3%), and several comorbidities (Table 1). Patients with self-reported sexual abuse had greater impairments in health status, exhibiting significantly lower VAS scores (53.1 (SD 21.2) versus 58.0 (SD 20.1), *P* = 0.03).

### 3.3. Independent Predictors of Sexual Abuse

**Figure 1:**
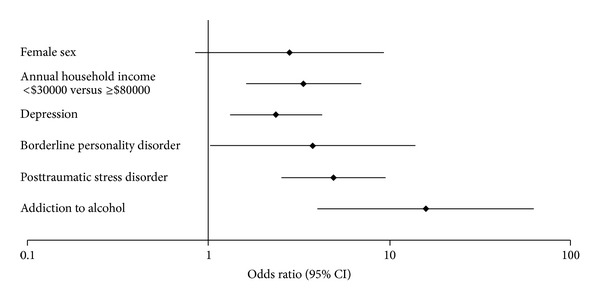
Significant predictors of sexual abuse: multivariable logistic regression analysis. Estimates adjusted for age, BMI, and all variables listed in the figure: female sex, annual household income of less than $30 000 compared to $80 000 or more, depression, borderline personality disorder, posttraumatic stress disorder, and addiction to alcohol.

Study-arm was not independently associated with abuse (*P* = 0.8) and was not included in the final model (Table 2). In the final model, age-, sex-, and BMI-adjusted independent predictors of sexual abuse were alcohol addiction (adjusted OR 15.8; 95% CI 4.0–62.8), posttraumatic stress disorder (4.9; 2.5–9.5), borderline personality (3.8; 1.0–13.8), depression (2.4; 1.3–4.3), and lower household income (3.4; 1.6–7.0) (Figure 1). The final model c-statistic was 0.79 (Table 2).

## 4. Discussion

In this study of 500 patients enrolled in a population-based regional obesity program, the prevalence of self-reported sexual abuse was 21.8%, and psychosocial issues such as addiction and psychiatric illnesses were the major independent predictors of abuse. Abused patients were more likely to be women than men, and they reported clinically important impairments in health status compared to those not abused.

The prevalence of sexual abuse in our study is within the range reported by other studies examining bariatric surgery candidates or recipients [12]. Our study differs from these in that we examined sexual abuse prevalence in a broader population (i.e., wait-listed patients, those undergoing intensive medical management and those awaiting bariatric surgery, all consecutively enrolled from a population-based, regional bariatric program). In the general population, recent studies examining the prevalence of sexual abuse have reported 4–20% prevalence rates, with a 13-fold higher risk in women [14–16]. Notably, several studies have shown that severely obese patients seeking bariatric surgery report about double the rate [12, 13, 23] of sexual abuse than these normative samples.

To our knowledge, only one other study has attempted to identify independent predictors of sexual abuse in a bariatric population [14]. This study found a 15.5% prevalence of sexual abuse in 573 bariatric surgery patients who were administered the PsyBari tool (which contains a single question on prior sexual abuse that was nearly identical to the item we used but measured in written fashion rather than using face-to-face interview) [14]. In women, independent predictors of sexual abuse were a history of prior physical abuse and prior suicidal ideation. In men, a history of prior psychiatric hospitalization was the only independent predictor. The author concluded that more studies are required to identify other independent predictors of abuse, including examination of additional psychosocial variables and comorbidities. Thus, our results extend and expand upon the work reported by Mahony. The clinically and sociodemographically rich nature of our data enabled us to assess the potential importance of many predictors and identify additional, previously unreported, independent predictors of abuse in a bariatric population. An additional strength of our study is the population-based nature of the data, as patients were enrolled from a large regional bariatric program.

The reported prevalence of sexual abuse varies according to the survey method used, the definition of sexual abuse (i.e., actual rape versus other types of inappropriate contact), the strength of the patient-researcher rapport, and the degree to which the patient may conceal, repress, or be unwilling to disclose past abusive experiences [13, 14]. Using the Childhood Trauma Questionnaire (CTQ) and face-to-face interviews produces higher rates of reported abuse [12]. Our approach, which involved a face-to-face single interview question, may have increased the identification of abuse compared to written questionnaires on one hand, but may have underestimated abuse compared to validated, focused abuse-related questionnaires on the other hand. One limitation to our methodology is that the term sexual abuse was not explicitly defined and there was no attempt to differentiate childhood sexual abuse from adult sexual abuse; we grouped all abusive experiences, whether past or current, into a single category. An additional limitation is that other predictor variables (such as psychiatric comorbidities or substance abuse) may have been underreported and, thus, their impact underestimated.

Low socioeconomic status, depression, addiction to alcohol, borderline personality disorder, and posttraumatic stress disorder were independent predictors of abuse. These findings are consistent with previous research demonstrating univariable associations between abuse and psychosocial pathology [7, 14]. However, we note that the literature shows inconsistencies with the association of sexual abuse and depression. Several studies have found higher depression scores in sexually abused patients [13, 14, 24, 25], and it has been suggested that a history of sexual abuse affects treatment outcomes by causing increased incidences of Axis 1 clinical disorders [24]. However, another study found no difference in depression scores at baseline between those who reported abuse and those who did not [26].

We also found that abused patients reported diminished health status compared to nonabused patients. The five-point mean difference in VAS scores is large enough to be clinically significant and the low VAS scores reflect a high degree of health impairment in this population (i.e., worse than VAS scores in type 2 diabetes and post-hip-fracture patients) [27]. Confirmation and further exploration of this finding is required; for example, a more detailed assessment of mental and physical health-related quality of life is warranted to more fully understand which domains of health are impaired.

What are the clinical implications, if any, of our work? If routine screening is not to be undertaken, we believe at the least those patients exhibiting one or more of the independent predictors for sexual abuse (especially addiction to alcohol given its very high odds ratio) should be specifically screened for abuse so that they may be offered supportive counseling or other treatments. The presence/absence of these other factors is often already documented during a standard medical history or medication review whereas sexual abuse screening is not commonly routinely performed. Previous studies examining the association between sexual abuse and weight loss after bariatric surgery have reported mixed results, and several of these analyses were underpowered. The preponderance of data suggests that sexual abuse is associated with lower early (i.e., after one year) postsurgical weight loss [5, 18]. Although weight loss outcomes at 2+ years may be similar between those abused and nonabused, high attrition rates limit the ability to draw definitive conclusions with longer-term data, and more study is needed [5, 18]. Because unresolved psychosocial stressors are felt to commonly contribute to a lack of successful medical weight management [12], there may be potential for improving weight management outcomes (either medical or surgical) by identifying and treating individuals with a prior history of abuse either before or concomitant with weight management interventions. This is a testable hypothesis. We should note that individuals with prior sexual abuse still exhibit substantial mean weight losses following bariatric surgery, and, thus, a history of abuse should not preclude performing surgery.

In conclusion, sexual abuse was common in the severely obese patients we studied and it affected their quality of life. Given the strong, independent associations between several psychosocial variables and sexual abuse, we recommend that these readily identifiable and high-risk patients be screened and offered supportive counseling or other treatments. Attention to these issues should improve weight-related outcomes and quality of life.

## Figures and Tables

**Table 1:** Baseline characteristics.

Variable	History of sexual abuse (*n* = 109) mean or *n*. (SD or %)	No history of sexual abuse (*n* = 391) mean or *n*. (SD or %)	*P* value
Female sex (%)	104 (95.4)	337 (86.2)	0.008
Mean age (years)	42.2 (9.2)	44.1 (9.7)	0.08
Weight (kg)	129.8 (23.4)	132.5 (25.5)	0.3
Body mass index (kg/m^2^)	47.8 (8.1)	47.9 (8.1)	0.8
Health status on visual analog scale	53.1 (21.2)	58.0 (20.1)	0.03

Marital status			0.003
Married/common-law	55 (50.5)	234 (59.9)	
Separated/divorced/widowed	30 (27.5)	54 (13.8)	
Single/never married	24 (22.0)	103 (26.3)	

Employment status			0.06
Full-time	60 (55.1)	254 (65.0)	
Part-time	13 (11.9)	51 (13.0)	
Other^1^	36 (33.0)	86 (22.0)	

Annual household income			<0.0001
Less than $30 000	28 (25.7)	40 (10.2)	
$30 000–$79 999	55 (50.5)	170 (43.5)	
$80 000 or greater	23 (21.1)	164 (41.9)	
Not answered	3 (2.8)	17 (4.4)	

Ethnicity			0.1
Caucasian	96 (88.1)	362 (92.6)	
Study arm			0.07
Medical	50 (45.9)	150 (38.4)	
Surgical	23 (21.1)	127 (32.5)	
Wait list	36 (33.0)	114 (29.2)	

Smoking status			0.4
Current smoker	12 (11.0)	37 (9.5)	
Former smoker	52 (47.7)	164 (41.9)	
Never smoked	45 (41.3)	190 (48.6)	

Hypertension	70 (64.2)	258 (66.0)	0.7
Dyslipidemia	36 (33.0)	130 (33.3)	0.9
Diabetes mellitus	41 (37.6)	141 (36.1)	0.7
Coronary artery disease	2 (1.8)	20 (5.1)	0.1
Sleep apnea	41 (37.6)	126 (32.2)	0.2
Nonalcoholic fatty liver disease	10 (9.2)	28 (7.2)	0.4
Gastroesophageal reflux disease	45 (41.3)	132 (33.7)	0.1
Asthma	40 (36.7)	86 (22.0)	0.002
Osteoarthritis	38 (34.9)	115 (29.4)	0.2
Polycystic ovary syndrome	21 (19.3)	42 (10.7)	0.02

Fibromyalgia	13 (11.9)	38 (9.7)	0.5
Depression	91 (83.5)	228 (58.3)	<0.0001
Bipolar and psychotic illness	16 (14.7)	13 (3.3)	<0.0001
Posttraumatic stress disorder	35 (32.1)	22 (5.6)	<0.0001
Binge eating disorder	44 (40.4)	104 (26.6)	0.005
Obsessive compulsive disorder	15 (13.8)	34 (8.7)	0.1
Addiction to drugs	10 (9.2)	9 (2.3)	0.003
Addiction to alcohol	13 (11.9)	3 (0.8)	<0.0001
Borderline personality disorder	14 (12.8)	5 (1.3)	<0.0001

^1^Home-maker, short-term disability, long-term disability, unemployed, retired, other (student), and casual/volunteer.

**Table 2:** Independent predictors of sexual abuse: multivariable logistic regression analysis.

Variable	Estimate (standard error)	Adjusted odds ratio (95% CI)
Age (years)	−0.008 (0.013)	0.99 (0.97–1.02)
Female sex	1.025 (0.613)	2.79 (0.84–9.27)
BMI (kg/m^2^)	0.010 (0.016)	1.01 (0.98–1.04)

Annual household income		
Less than $30 000 versus $80 000 or greater	1.208 (0.376)	3.35 (1.60–6.99)
$30 000–$79 999 versus $80 000 or greater	0.665 (0.297)	1.94 (1.09–3.48)
Not answered versus $80 000 or greater	0.059 (0.740)	1.06 (0.25–4.52)

Posttraumatic stress disorder	1.585 (0.338)	4.88 (2.52–9.46)
Addiction to alcohol	2.762 (0.703)	15.8 (3.99–62.8)
Depression	0.857 (0.302)	2.36 (1.30–4.26)
Borderline personality disorder	1.320 (0.665)	3.75 (1.02–13.8)

Model c-statistic = 0.79.
